# Natural genetic variation in dynamic photosynthesis is correlated with stomatal anatomical traits in diverse tomato species across geographical habitats

**DOI:** 10.1093/jxb/erae082

**Published:** 2024-04-12

**Authors:** Yugo Yoshiyama, Yu Wakabayashi, Kristin L Mercer, Saneyuki Kawabata, Takayuki Kobayashi, Toshihito Tabuchi, Wataru Yamori

**Affiliations:** Graduate School of Agricultural and Life Sciences, The University of Tokyo, Nishitokyo, Tokyo, Japan; Graduate School of Agricultural and Life Sciences, The University of Tokyo, Nishitokyo, Tokyo, Japan; Graduate School of Agricultural and Life Sciences, The University of Tokyo, Nishitokyo, Tokyo, Japan; Ohio State University, Department of Horticulture and Crop Science, Columbus, OH, USA; Graduate School of Agricultural and Life Sciences, The University of Tokyo, Nishitokyo, Tokyo, Japan; Department of Advanced Food Sciences, College of Agriculture, Tamagawa University, Machida, Tokyo, Japan; Department of Advanced Food Sciences, College of Agriculture, Tamagawa University, Machida, Tokyo, Japan; Graduate School of Agricultural and Life Sciences, The University of Tokyo, Nishitokyo, Tokyo, Japan; University of Essex, UK

**Keywords:** Natural variation, photosynthetic induction, stomatal conductance, stomatal density, stomatal size, water use efficiency

## Abstract

Plants grown under field conditions experience fluctuating light. Understanding the natural genetic variations for a similarly dynamic photosynthetic response among untapped germplasm resources, as well as the underlying mechanisms, may offer breeding strategies to improve production using molecular approaches. Here, we measured gas exchange under fluctuating light, along with stomatal density and size, in eight wild tomato species and two tomato cultivars. The photosynthetic induction response showed significant diversity, with some wild species having faster induction rates than the two cultivars. Species with faster photosynthetic induction rates had higher daily integrated photosynthesis, but lower average water use efficiency because of high stomatal conductance under natural fluctuating light. The variation in photosynthetic induction was closely associated with the speed of stomatal responses, highlighting its critical role in maximizing photosynthesis under fluctuating light conditions. Moreover, stomatal size was negatively correlated with stomatal density within a species, and plants with smaller stomata at a higher density had a quicker photosynthetic response than those with larger stomata at lower density. Our findings show that the response of stomatal conductance plays a pivotal role in photosynthetic induction, with smaller stomata at higher density proving advantageous for photosynthesis under fluctuating light in tomato species. The interspecific variation in the rate of stomatal responses could offer an untapped resource for optimizing dynamic photosynthetic responses under field conditions.

## Teaser

The response of stomatal conductance plays a pivotal role in photosynthetic induction, with smaller stomata at higher density proving advantageous for photosynthesis under fluctuating light conditions in tomato species.

## Introduction

Tomato (Solanaceae) is one of the most consumed horticultural crops worldwide (Ntonifor *et al*., 2013). The *Solanum* genus includes the cultivated tomato [*Solanum lycopersicum* L. var. *lycopersicum* and *S. lycopersicum* var. *cerasiforme* (cherry tomato)] and its 12 closest wild relatives [*S. pimpinellifolium* and *S. lycopersicum* var. *cerasiforme* (crop progenitors), as well as *S. arcanum*, *S. cheesmaniae*, *S. chilense*, *S. chmielewskii*, *S. corneliomulleri*, *S. galapagense*, *S. habrochaites*, *S. huaylasense*, *S. neorickii*, *S. pennellii*, and *S. peruvianum*] (Peralta *et al*., 2008; Knapp and Peralta, 2016). The wild tomatoes are native to western South America and are found across diverse habitats, from the coastal deserts of the Pacific at sea level, to the verdant inter-Andean valleys, to the mountainous Andean regions at altitudes of up to 3300 m a.s.l. (Warnock, 1991). The global human population is projected to reach 9.5 billion by 2050, requiring a 60% increase in crop production to satisfy the rapidly growing demand for staple foods (Godfray *et al*., 2010) and the nutritional needs of a healthy diet (Ray *et al*., 2013). Thus, further enhancements in crop yield are imperative.

Numerous strategies have been used to breed for higher crop yields. Some have suggested improving leaf photosynthetic capacity as a promising target since it is the fundamental factor determining crop yields by converting light energy into biomass (Long *et al*., 2006; Yamori *et al*., 2016; Qu *et al*., 2021). Until recently, efforts to improve photosynthetic performance have focused on steady-state conditions and, consequently, crop breeding programs have often overlooked the dynamic response of photosynthesis to fluctuating light conditions encountered in the field. However, there is now a growing recognition of the importance of photosynthetic performance under fluctuating light as a key target for enhancing crop yields (e.g. Zhu *et al*., 2004; Yamori and Shikanai, 2016; Taylor and Long, 2017; Murchie *et al*., 2018; Papanatsiou *et al*., 2019). When light intensity suddenly increases, it requires 10–30 min for the photosynthetic rate to stabilize (Pearcy, 1990; Lawson *et al*., 2012; McAusland *et al*., 2016; Yamori, 2016). This process, known as the photosynthetic induction response, has the potential to limit photosynthesis under fluctuating light conditions. Simulation analyses have revealed that the potential loss of daily carbon gain due to photosynthetic induction can exceed 20% in soybean [*Glycine max* (L.) Merr.] (Tanaka *et al*., 2019) and wheat (*Triticum aestivum* L.) (Taylor and Long, 2017) under conditions simulating natural light fluctuations in the field.

The most important genetic resource for breeders is novel genetic variation for traits of interest found in plants compatible with the crop. Despite concerns of introducing linkage drag, wild relatives of crops can contain genetic variants that can be beneficial when introgressed into crop varieties (Tanksley and McCouch, 1997; Bohra *et al.*, 2022). Wild relatives have often been used to contribute tolerance to biotic and abiotic factors (e.g. in tomato: Schouten *et al.*, 2019) since wild species have evolved over time under diverse conditions and may be locally adapted to their environments of origin (Kawecki and Ebert, 2004; Wadgymar *et al.*, 2022). Since the geographic range of each species can encompass multiple climates, individual accessions from a species may have become adapted over time to particular environmental conditions (Wadgymar *et al.*, 2022). Relevant wild germplasm may be sourced from climates that imposed natural selection on useful adaptations (Redden, 2013). Exploring natural genetic variation for photosynthetic efficiency may significantly contribute to effective breeding programs (Tanaka *et al*., 2019). For instance, research using quantitative trait locus analysis and genome-wide association studies has identified chromosomal regions associated with intraspecific variation in steady-state photosynthesis among various crops, leading to improvements in photosynthesis through marker-assisted selection (Takai *et al*., 2013; Sakoda *et al*., 2020a; Yang *et al*., 2020).

Recently, breeders have proposed the use of natural genetic variation in dynamic photosynthesis under fluctuating light conditions (Lawson *et al*., 2012; Tanaka *et al.*, 2019). Yet our understanding of these dynamic responses lags behind that of steady-state photosynthesis. Reports highlight interspecific and intraspecific variations in photosynthetic induction in rice (Qu *et al*., 2016; Adachi *et al*., 2019a; Acevedo-Siaca *et al*., 2020; Taniyoshi *et al*., 2020), wheat (Faralli *et al*., 2019b), soybean (Soleh *et al*., 2016a, b), cassava (De Souza *et al*., 2020), sorghum (Pignon *et al*., 2021), and banana (Eyland *et al*., 2021b). Also, a comparison of photosynthetic induction in tomato cultivars highlights the importance of manipulating stomatal traits for speeding up photosynthetic induction (Zhang *et al*., 2022). Furthermore, in a comparative study between wild tomatoes and *flacca* mutant tomatoes with less abscisic acid (ABA), mutant tomatoes overcome a substantial limitation on non-steady-state photosynthesis through stomata that remain open in low light (Kaiser *et al*., 2020). However, natural genetic variation for dynamic photosynthesis with stomatal anatomical traits has yet to be explored in diverse tomato species sourced across geographical and climatic gradients that may have selected differentially on photosynthesis-related traits. Such information would be vital for devising strategies to enhance carbon gain in tomato under natural field conditions.

Recent research using mutants or transgenic plants has shown that the rate of stomatal opening can limit photosynthetic induction (Papanatsiou *et al*., 2019; Shimadzu *et al*., 2019; Kimura *et al*., 2020; Yamori *et al*., 2020). Stomata regulate CO_2_ assimilation and water loss via transpiration. A rapid response of stomatal conductance to fluctuating light can optimize both photosynthetic efficiency and water use efficiency (WUE) (Lawson *et al*., 2010, 2012). Recent studies have focused on the relationship between stomatal morphology and dynamic photosynthesis, but observations conflict. Some studies have reported that both stomatal density and size influence the light-induced kinetics of stomatal conductance (Hetherington and Woodward, 2003; Drake *et al*., 2012; Raven, 2014; Vialet-Chabrand *et al*., 2017; Sakoda *et al*., 2020b), while others have found either no correlation or only a weak inter- and intraspecific correlation between stomatal anatomy and the light-induced kinetics of stomatal conductance (McAusland *et al*., 2016; Papanatsiou *et al*., 2016; Schuler *et al*., 2017; Faralli *et al*., 2019a; Eyland *et al*., 2021b). These results may imply that stomatal anatomy, or molecular mechanisms of stomatal opening and closing (e.g. H^+^-ATPase), may limit light-induced kinetics of stomatal conductance. The mechanism responsible in any given case might vary among plant species. Furthermore, to the best of our knowledge, there are no reports demonstrating interspecific variation in the rate of stomatal responses in tomato.

Here, we revealed genetic diversity in photosynthetic induction and light-induced stomatal dynamics in relation to stomatal anatomy across nine tomato species under both steady-state and fluctuating light conditions. We posed three main questions. (i) Is there significant diversity not only in steady-state photosynthesis, but also in dynamic photosynthetic responses, under non-steady-state conditions of fluctuating light among tomato species? (ii) Are interspecific differences in photosynthesis under steady-state conditions consistent with those observed under non-steady-state conditions of fluctuating light? (iii) What are the primary factors driving natural variations in photosynthesis, stomatal conductance, and intrinsic WUE (iWUE) in relation to stomatal anatomical features? By comparing photosynthetic induction and light-induced stomatal dynamics with stomatal traits, we seek insights into the significance of stomatal responses in daily carbon gain and iWUE. Such variations can serve as a valuable foundation for genetically enhancing dynamic photosynthesis and crop yields in field environments.

## Materials and methods

### Plant materials

**Fig. 1.**
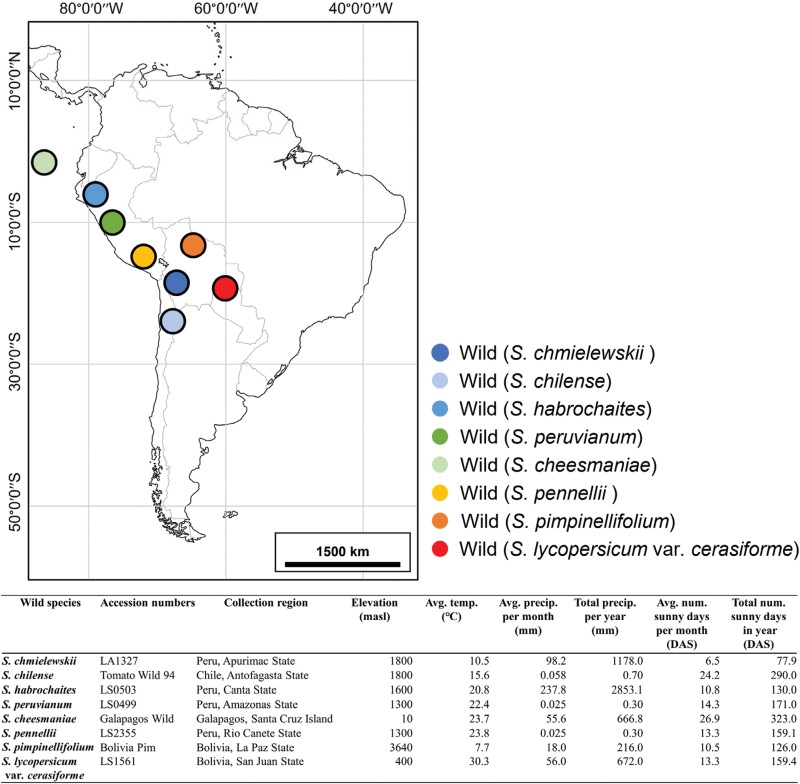
Map of the locations in South America where eight accessions from eight wild tomato species were collected, and table of the climatic data associated with those collection points. Climatic data from the World Meteorological Organization (WMO) for 2016–2022.

We grew two cherry tomato cultivars (*Solanum lycopersicum* L. var. *cerasiforme* called ‘Chika’ and ‘Orange Chika’; Takii and Co., Ltd, Kyoto, Japan) and eight wild tomato species: *S. lycopersicum* var. *cerasiforme* (accession no. LS1561), *S. chmielewskii* (LA1327), *S. chilense* (‘Tomato Wild 94’), *S. habrochaites* (LS0503), *S. pennellii* (LS2355), *S. pimpinellifolium* (‘Bolivia Pim’), *S. peruvianum* (LS0499), and *S. cheesmaniae* (‘Galapagos Wild’). The locations in South America, where eight accessions from eight wild tomato species were collected, have been summarized with the climatic data associated with those collection points (Fig. 1). Seeds were sown in peat moss, and seedlings were transferred to rockwool cubes (10 cm×10 cm×6.5 cm, Grodan Delta Blocks) upon unfolding of the second true leaf. Plants were grown in a controlled-environment glasshouse with natural radiation and ambient CO_2_ partial pressure from 7 August to 7 November 2021 at the University of Tokyo (35°43ʹN, 139°32ʹE). In the environmental setting of the glasshouse, air was constantly circulated by ventilation fans, and skylights were opened when the temperature was >25 °C. The average air temperature and relative humidity in the glasshouse during the growing period were 24.6 °C and 58.3%, respectively. Plants were regularly irrigated with modified GG nutrient solution (Eco-Guerrilla Company: EC=1.0 ± 0.05 dS m^−2^ N=0.8%, P=1.4%, K=5.25%) and randomly rotated daily to avoid position effects. The experiment was conducted in a completely randomized design with four biological replicates (individual plants).

### Photosynthetic induction and diurnal change of photosynthesis

The photosynthetic induction response was measured on the youngest, fully expanded leaf, the fifth leaf from the stem apex, using a portable gas exchange system (LI-6400XT; Li-Cor, Lincoln, NE, USA). Measurements were made at the center leaf lamina, avoiding the main veins. Plants were selected at random and measured from 07:00 h to 14:00 h to avoid confounding species with time of day and to minimize any diurnal influences. To evaluate photosynthetic induction, plants were dark adapted for at least 1 h before measurements. Before the measurements, the leaf was acclimatized to the conditions of the LI-6400XT cuvette for 5 min in the dark at a CO_2_ concentration of 400 µmol mol^−1^, an air temperature of 25 °C, relative humidity of 65%, and a flow rate of air through the system of 400 µmol s^–1^. After stomatal conductance (*g*_s_), photosynthetic rate (*A*), and intercellular CO_2_ concentration (*C*_i_) had steadied, gas exchange was measured in the dark for 5 min, then the light was increased in a single step to 1500 µmol m^−2^ s^−1^, which was maintained for 50 min. Although such a dark–light transition rarely occurs in nature, we chose this extreme transition to maximize the effects on the photosynthetic induction response (Allen and Pearcy, 2000; Urban *et al*., 2007; Guo *et al*., 2016; Kaiser *et al*., 2017; Zhang *et al*., 2018). Photosynthetic parameters were recorded every 30 s.

The maximum photosynthetic rate (*A*_max_) and maximum stomatal conductance (*g*_smax_) were considered to correspond to steady-state photosynthesis. We calculated the time to reach 50, 80, and 90% of maximum (*t*50_*A*_, *t*50_*g*s_; *t*80_*A*_, *t*80_*g*s_; and *t*90_*A*_, *t*90_*g*s_). Stomatal conductance at the beginning of induction (*g*_sinitial_) was the last value obtained before the light was increased to 1500 µmol m^−2^ s^−1^. Photosynthetic induction (PI) was calculated as a percentage of the total change between initial *A* (*A*_i_) and final *A* (*A*_f_) of each transient: PI=(*A*–*A*_i_)/(*A*_f_–*A*_i_).

The rate of CO_2_ assimilation without stomatal limitation (A*) was calculated as: $A^{*}=\left\{(A+R_{d})(C_{i}f-\Gamma^{*})/(C_{i}-\Gamma^{*})-R_{d}\right\}$, where *C*_if_ is the final *C*_i_ (*C*_i_ at the end of the induction period) and *R*_d_ is the dark respiration rate. Subsequently, transient stomatal limitation (*L*_S_) and transient biochemical limitation (*L*_B_) during photosynthetic induction were calculated (Allen and Pearcy, 2000; Urban *et al*., 2007; Kaiser *et al*., 2017) as $L_{S}=\left\{({A^{*}}_{Ca}-A)/(A_{f}-A_{i})\right\}\times 100$; and $L_{B}=\left\{(A_{f}{-}^{\circ }{A^{*}}_{Ci})/(A_{f}-A_{i})\right\}\times 100$, where A*_Ca_ and A*_Ci_ are *A* corrected for changes in transient *C*_i_. In this equation, stomatal limitations to photosynthesis are removed by recalculating the photosynthetic rate to a constant *C*_i_.

We also examined photosynthetic responses to more realistic fluctuating light (Kimura *et al*., 2020). This fluctuating light reproduced the outdoor light at a daylength of 10 h, and the light intensity varied every 10 s in the range of a maximum value of 1250 µmol m^−2^ s^−1^ and a minimum value of 31 µmol m^−2^ s^−1^. Changes in irradiance were measured every 10 s in a rice paddy field in Kyoto Prefecture in Japan (35°02ʹN, 135°47ʹE), for 12 h on 23 July 2016 and on the floor of a deciduous broad-leaved forest in Kanagawa Prefecture (35°21ʹN, 139°16ʹE) for ~2 h around midday on 28 April 2017. The light fluctuation was reproduced at an air temperature of 25 °C and relative humidity of 65% in a portable gas exchange system (LI-6400XT), and the photosynthetic parameters were recorded every 10 s. We calculated total photosynthetic induction $\left[A_{SUM}=\sum A\left(t\right)\right]$, average stomatal conductance (*g*_sAVE_), and average iWUE (iWUE_AVE_:WUE(*t*)=*A*(*t*)/*g*_s_(*t*)].

### Stomatal traits

Leaf samples on which to measure the stomatal size, density, and index were taken on the same leaf used for gas exchange measurements. Stomatal size, density, and index were measured in four randomly selected fields of view (four technical replicates) of both the adaxial and abaxial surfaces of four detached leaves of each species under an electron microscope (JCM-6000, JEOL, Tokyo, Japan). For each sample, the imaging of stomatal characteristics by the electron microscope was completed within 3 min to avoid excessive cell shrinkage caused by the vacuum. Stomatal number was counted to calculate stomatal density (SD, mm^−2^). Stomatal index (SI, %) was calculated as SI=SD/(SD+ECD)×100, where ECD is the epidermal cell density (mm^−2^). We assumed stomata to be elliptical when measuring their size, including guard cell area (Savvides *et al*., 2012). Stomatal size was considered to be the long diameter portion of an ellipse, and measured by Image J v. 1. 53 software.

### Statistical analysis

The Tukey–Kramer method was used to evaluate differences in photosynthetic and stomatal parameters between species. Pearson’s correlation coefficients were calculated, and the significance of relationships was tested by two-tailed *t*-tests (*P*<0.05). All statistical analyses were performed in R v. 4.3.2 or SAS v9.4 software.

## Results

### Photosynthetic induction responses vary significantly among tomato species

**Fig. 2.**
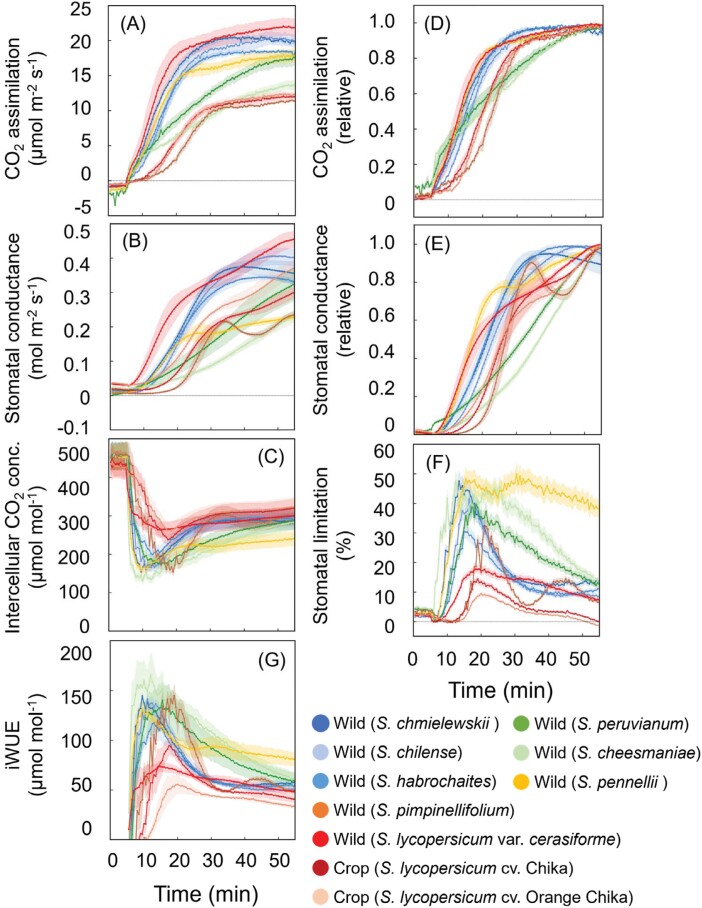
Photosynthetic induction in eight wild tomato species and two cherry tomato cultivars. (A–C) CO_2_ assimilation rate, stomatal conductance, and intercellular CO_2_ concentration; (D, E) normalized CO_2_ assimilation and stomatal conductance; (F) stomatal limitation (*L*_S_) throughout photosynthetic induction: *L*_B_={(*A*_f_−*A*_*C*i_*)/(*A*_f_ − *A*_i_)}×100, where *A*_*C*a_* and *A*_*C*i_* are *A* corrected for changes in transient intercellular CO_2_ concentration; and (G) intrinsic water use efficiency (iWUE) throughout irradiation. Data are means ±SE, *n*=4. See text for abbreviations.

The induction of photosynthesis (*A*) and stomatal conductance (*g*_s_) showed biphasic responses to the change from dark to high light (Fig. 2A, B). We considered the maximum photosynthetic rate (*A*_max_) and the maximum stomatal conductance (*g*_smax_) of each induction curve to correspond to steady-state photosynthesis. *A*_max_ and *g*_smax_ differed significantly among species (*A*_max_, 11.6–22.1 µmol m^−2^ s^−1^; *g*_smax_, 0.232–0.455 mol m^−2^ s^−1^), being highest in *S. lycopersicum* var. *cerasiforme* and lowest in *S. pimpinellifolium* and *S. cheesmaniae* (Supplementary Fig. S1A, E). To clarify the differences in photosynthetic induction among species, we normalized *A* and *g*_s_ by regarding minimum values as 0.0 and maximum values as 1.0 throughout inductions (Fig. 2D, E). *t*50_*A*_ and *t*50_*g*s_ differed significantly among species (*t*50_*A*_, 13.3–22.5 min; *t*50_*g*s_, 17.2–36.7 min; Supplementary Fig. S1B, F). *t*80_*A*_, *t*80_*g*s_, *t*90_*A*_, and *t*90_*g*s_ responded similarly (Supplementary Fig. S1C, D, G, H). There were significant positive correlations between *t*50_*A*_ and *t*80_*A*_ or *t*90_*A*_, and between *t*50_*g*s_ and *t*80_*g*s_ or *t*90_*g*s_ (Supplementary Fig. S2). The *A* and *g*_s_ were induced fastest in *S. lycopersicum* var. *cerasiforme*, *S. chmielewskii*, and *S. pennellii*, and slowest in *S. pimpinellifolium*, *S. peruvianum*, and *S. cheesmaniae* (Fig. 2A, B). As a result, the response of *C*_i_ varied greatly among species (Fig. 2C). In addition, the value of *g*_sinitial_ differed among species (Supplementary Fig. S1I). Thus, species differed significantly in photosynthetic traits in both steady-state and non-steady-state responses to changing light conditions.

We calculated stomatal limitation (*L*_S_) and biochemical limitation (*L*_B_) to evaluate the reductions in photosynthesis (%) due to limiting factors during induction, compared with that at steady state. At the start of photosynthetic induction, *L*_B_ contributed almost 100% to the limitation of photosynthesis, but, with increasing irradiation, *L*_B_ gradually decreased while *L*_S_ gradually increased from 0% to 10–50% over the first 20 min (Supplementary Fig. S3). This indicated that stomatal limitation during the induction response was significant in most species (Fig. 2F). The extent of *L*_S_ during photosynthetic induction varied among cultivars (Fig. 2F). *L*_S_ was lowest in the two tomato cultivars, as can be seen in the smaller drop of *C*_i_ at the start of photosynthetic induction; the other species tended to suffer more from *L*_S_, as can be seen in the large drop of *C*_i_ in the initial phase (Fig. 2C). *L*_S_ was highest in *S. pennellii*, *S. peruvianum*, and *S. cheesmaniae*, especially at the later phase of induction, after 20 min (Fig. 2F; Supplementary Fig. S3).

### Relationships between photosynthetic induction and steady-state photosynthesis

**Fig. 3.**
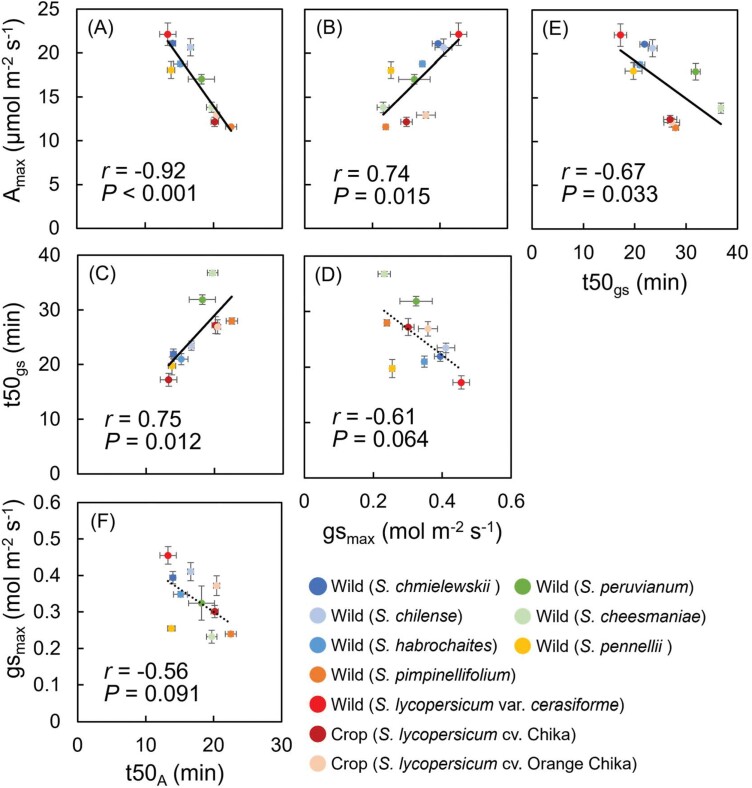
Correlations between parameters of steady-state photosynthesis (maximum photosynthetic rate, *A*_max_; maximum stomatal conductance, *g*_smax_) and photosynthetic induction (time to reach 50% of maximum rate, *t*50_*A*_ and *t*50_*g*s_). (A–F) Correlations between (A) *A*_max_ and *t*50_*A*_, (B) *A*_max_ and *t*50_*g*s_, (C) *t*50_*g*s_ and *t*50_*A*_, (D) *t*50_*g****s***_ and *g*_smax_, (E)*g*_smax_ and *t*50_*g*s_, and (F) *A*_max_ and *t*50_*g*s_. Data are means ±SE, *n*=4. Solid lines indicate significant correlations. See text for abbreviations.

We analyzed relationships between parameters of photosynthetic induction and steady-state photosynthesis (Fig. 3). There was a significant negative correlation between *A*_max_ and the rate of induction (Fig. 3A). There were significant positive correlations between *A*_max_ and *g*_smax_ (Fig. 3B) and between *t*50_*A*_ and *t*50_*g*s_ (Fig. 3C), but there was no significant correlation between *g*_smax_ and *t*50_*g*s_ (Fig. 3D), or between *g*_sinitial_ and *t*50_*A*_ (*r*= −0.058, *P*>0.80; Supplementary Fig. S1) or *t50*_*g*s_ (*r*=−0.14, *P*>0.70; Supplementary Fig. S1).

### Relationships between photosynthetic induction and diurnal changes of photosynthetic traits

**Fig. 4.**
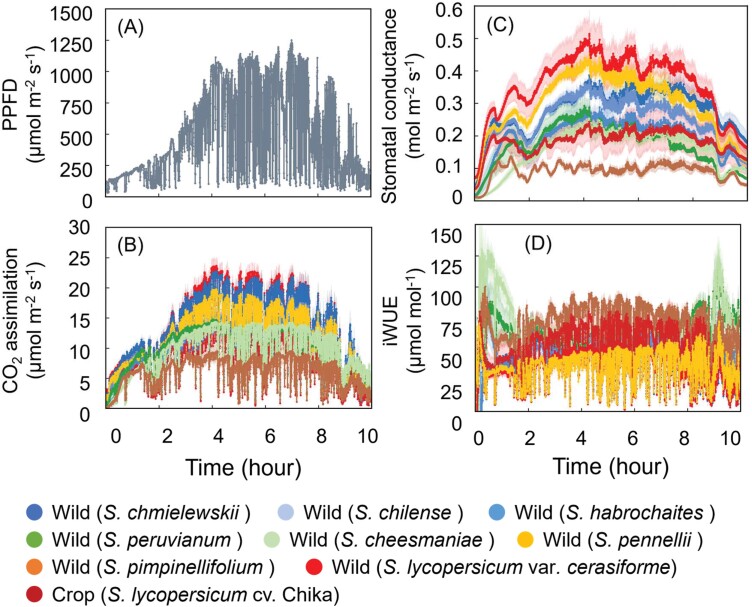
Photosynthetic parameters under simulated natural fluctuating light for 10 h. (A) Light intensity of irradiation. (B) CO_2_ assimilation rate throughout irradiation. (C) Stomatal conductance throughout irradiation. (D) Intrinsic water use efficiency (iWUE) throughout irradiation. Data are means ±SE, *n*=4.

To evaluate the effect of *g*_s_ responses on diurnal changes of photosynthetic traits (*A*, *g*_s_, and iWUE), we subjected plants to fluctuating light intensities over an entire diurnal period (Fig. 4A). Throughout the period, *g*_s_ of *S. pimpinellifolium*, *S. peruvianum*, and *S. cheesmaniae* appeared to be lower than those of the two tomato cultivars (Fig. 4C), as observed in the *g*_s_ response during photosynthetic induction (Fig. 2B). Species that opened their stomata in the morning also opened them in the afternoon, resulting in higher photosynthesis during the day (Fig. 4B). iWUE varied significantly across species (Fig. 4D); plants with lower *g*_s_ tended to have greater iWUE (Fig. 4C, D). Under low light in the early morning and late afternoon, the extent of *A* increase was larger than that of *g*_s_ increase, especially in *S. pimpinellifolium*, *S. peruvianum*, and *S. cheesmaniae*, resulting in greater iWUE than in the two tomato cultivars (Fig. 4D). *A*_SUM_, *g*_sAVE_, and iWUE_AVE_ varied significantly among species (Supplementary Fig. S4).

**Fig. 5.**
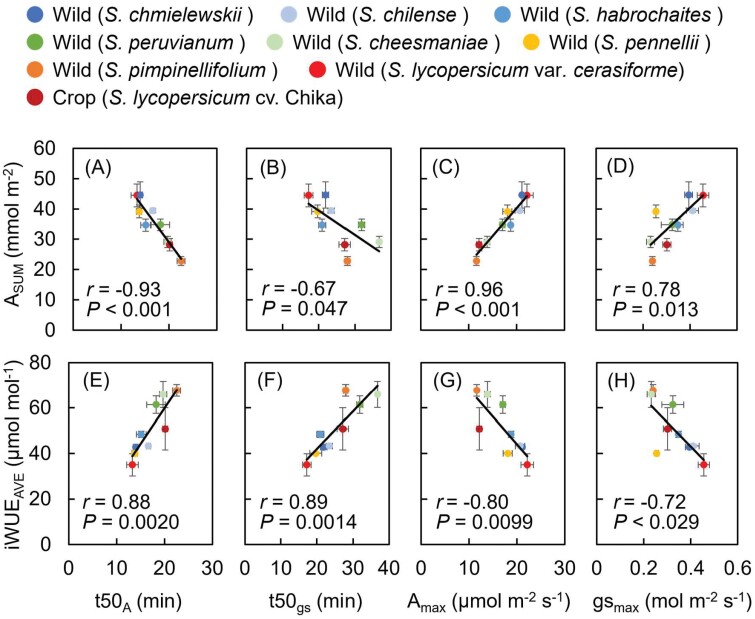
Correlations between parameters of diurnal response of photosynthesis (total photosynthetic induction, *A*_SUM_; average intrinsic water use efficiency, iWUE_AVE_; steady-state photosynthesis, *A*_max_ and *g*_smax_; and photosynthetic induction, *t*50_*A*_ and *t*50_*g*s_). (A–H) Correlations of (A, B) *A*_SUM_ with photosynthetic induction parameters *t*50_*A*_ and *t*50_*g*s_; (C, D) *A*_SUM_ with steady-state photosynthesis parameters *A*_max_ and *g*_smax_; (E, F) iWUE with *t*50_*A*_ and *t*50_*g****s***_; (G, H) iWUE with *A*_max_ and *g*_smax_. Data are means ±SE, *n*=4. Solid lines indicate significant correlations. See text for abbreviations.

Next, we compared the relationship between photosynthetic performance during photosynthetic induction (Fig. 2) and diurnal change of photosynthesis (Fig. 4) using Pearson’s *r*. Among induction traits, there were significant positive correlations between *A*_SUM_ and *A*_max_, *A*_SUM_ and *g*_smax_, iWUE_AVE_ and *t*50_*A*_, and iWUE_AVE_ and *t*50_*g*s_ (Fig. 5C–F), and significant negative correlations between *A*_SUM_ and *t*50_*A*_, *A*_SUM_ and *t*50_*g*s_, iWUE_AVE_ and *A*_max_, and iWUE_AVE_ and *g*_smax_ (Fig. 5A, B, G, H).

### Natural variation in stomatal anatomy

**Fig. 6.**
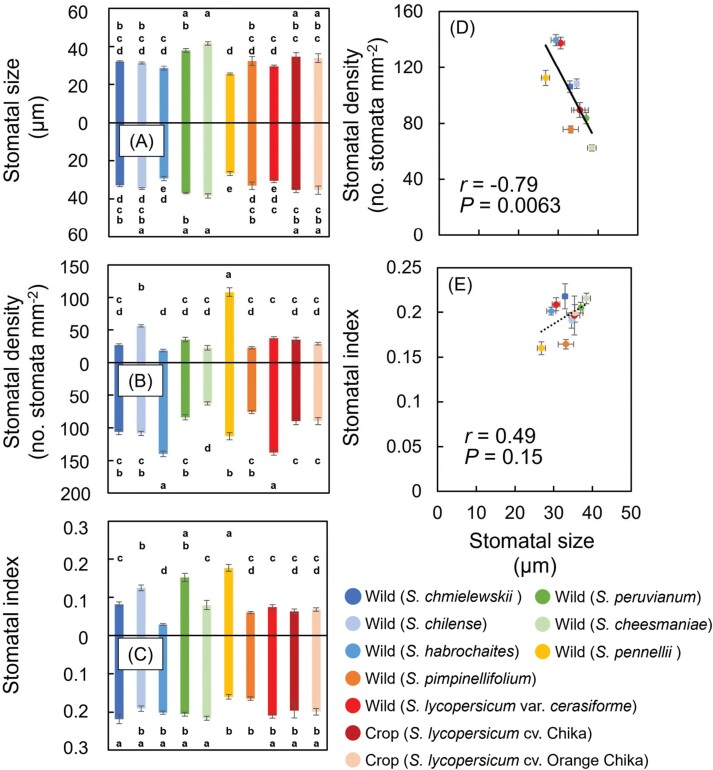
Stomatal characteristics. (A–C) Stomatal size, density, and index. Positive *y*-axis, adaxial side; negative *y*-axis, abaxial side. (D, E) Correlations between stomatal density and stomatal size on the (D) abaxial and (E) abaxial side. Data are means ±SE, *n*=4. Bars with the same letter are not significantly different at *P*<0.05 by Tukey’s test. Solid line indicates significant correlations.

We quantified stomatal size, density, and index on both abaxial and adaxial sides. Stomatal features varied greatly among species (Fig. 6). Stomatal size was similar on both leaf sides in all species, and ranged from 25.5 μm to 38.8 μm (Fig. 6A). Stomatal density ranged from 62.5 mm^–2^ to 139.6 mm^−2^ on the abaxial side and from 18.8 mm^–2^ to 108.3 mm^−2^ on the adaxial side (Fig. 6B). Stomatal index ranged from 0.16 to 0.22 on the abaxial side and from 0.029 to 0.18 on the adaxial side (Fig. 6C). In *S. pennellii*, stomata were evenly distributed on both sides, while in the other species, they were more abundant on the abaxial side (Fig. 6B). Stomatal size was smaller in *S. pennellii* than in the two tomato cultivars, but was not significantly different in the other species. On the other hand, some species had smaller values and others had larger values than in the two tomato cultivars (Fig. 6A). Stomatal size was not correlated with stomatal index, but it was negatively correlated with stomatal density on both the adaxial and abaxial sides (Fig. 6D, E).

### Relationships between stomatal anatomy and light-induced stomatal dynamics

**Fig. 7.**
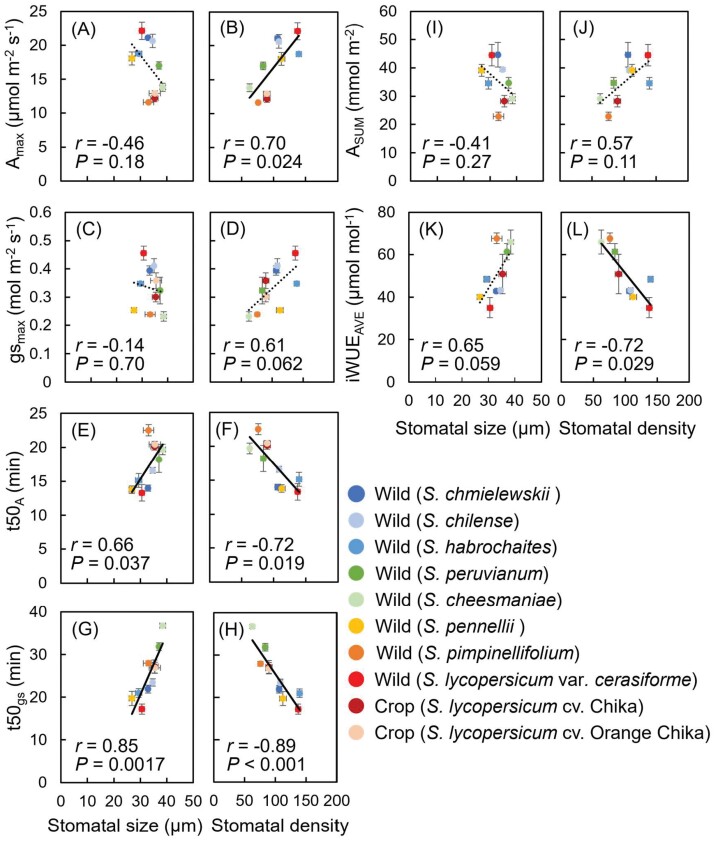
Correlations of stomatal anatomical characteristics on the abaxial side with photosynthetic parameters. (A, C, E, G) Stomatal size with photosynthetic induction parameters *A*_max_, *g*_smax_, *t*50_*A*_, and *t*50_*g*s_. (B, D, F, H) Stomatal density with *A*_max_, *g*_smax_, *t*50_*A*_, and *t*50_*g*s_. (I, K) Stomatal size with diurnal irradiation parameters *A*_SUM_ and iWUE_AVE_. (J, L) Stomatal density with *A*_SUM_ and iWUE_AVE_. Data are means ±SE, *n*=4. Solid lines indicate significant correlations. See text for abbreviations.

We analyzed the relationships between stomatal size and density and photosynthetic performance (Fig. 7). There was no relationship between stomatal characteristics with steady-state parameters (*A*_max_ and *g*_smax_) (Fig. 7A, C, D), except for the relationship between *A*_max_ and stomatal density (Fig. 7B). On the other hand, there were significant positive correlations of stomatal size and significant negative correlations of stomatal density at the abaxial side or at both the abaxial and adaxial sides with *t*50_*A*_ and *t*50_*g*s_ (Fig. 7E–H; Supplementary Fig. S5). Moreover, there was no significant relationship of stomatal size but there were significant relationships of stomatal density with iWUE (Fig. 7L).

## Discussion

Improving photosynthesis under fluctuating light conditions is important to sustaining or further increasing crop yields. Exploring natural genetic variation can contribute to developing effective breeding programs for photosynthesis. Our results show the following. (i) We found interspecific variation in both steady-state and non-steady-state *A* and *g*_s_, as well as in stomatal size and density, among eight wild tomato species and two tomato cultivars. (ii) For both *A* and *g*_s_, we also identified positive correlations between measurements under steady-state and non-steady-state fluctuating light conditions. (iii) Finally, we found that the smaller the stomatal size and the higher the stomatal density, the faster is the photosynthetic induction. In particular, the higher the stomatal density, the higher the integrated photosynthesis under simulated natural fluctuating light over 10 h. Moreover, some wild tomato species had greater photosynthetic performance in both steady-state and non-steady-state conditions than the two tomato cultivars, indicating great potential to utilize wild materials to improve photosynthesis in cultivated tomatoes for improvement of crop yields. On the other hand, since there is a trade-off between high photosynthesis and iWUE (Fig. 5E, G), it may be necessary to prioritize either iWUE or photosynthesis when improving cultivated species.

### Stomatal behavior varies widely during light fluctuations, greatly limiting photosynthesis

We found interspecific variations in photosynthetic parameters under steady-state (*A*_max_, *g*_smax_: Fig. 2A, B; Supplementary Fig. S1A, E) and non-steady-state conditions (*t*50_*A*_, *t*50_*g*s_: Fig. 2A, B; Supplementary Fig. S1B, F); in stomatal size and density (Fig. 6A, B); and in integrated photosynthesis and iWUE under simulated natural fluctuating light (*A*_SUM_, iWUE_AVE_: Fig. 4; Supplementary Fig. S4). All relationships are summarized in a heatmap (Supplementary Fig. S6). Previous studies have reported variation in the photosynthetic induction response both between species (Allen and Pearcy, 2000; Tomimatsu and Tang, 2012; Wong *et al*., 2012; Takagi *et al*., 2017; Wachendorf and Küppers, 2017; Xiong *et al*., 2018) and within species (e.g. Arabidopsis, rice, soybean, and wheat) (Alter *et al*., 2012; Qu *et al*., 2016; Soleh *et al*., 2016a, b; Faralli *et al*., 2019b; Salter *et al*., 2019; Acevedo-Siaca *et al*., 2020; Taniyoshi *et al*., 2020). To our knowledge, this is the first report demonstrating natural variation in photosynthetic induction and the light-induced stomatal dynamics in tomatoes.

Species with faster *g*_s_ opening responses (e.g. *S. lycopersicum* var. *cerasiforme*, *S. habrochaites*, and *S. pennellii*) achieved 50% *A* more rapidly than others with slower *g*_s_ kinetics (e.g. *S. cheesmaniae*, *S. peruvianum*, and *S. pimpinellifolium*), as supported by the positive correlation between *t*50_*A*_ and *t*50_*g*s_ (Fig. 3C). These findings corroborate previous reports that identified speedy stomata’s characteristics as a potential target for optimizing CO_2_ diffusion and dynamic photosynthesis under fluctuating light. A higher steady-state *g*_s_ has been linked with faster light-induced *g*_s_ responses (Drake *et al*., 2012; Kaiser *et al*., 2016; McAusland *et al*., 2016; Wachendorf and Küppers, 2017; Sakoda et al., 2020b), although Eyland *et al*. (2021b) reported no relationship between steady-state *g*_s_ values and light-induced *g*_s_ response. Also, a previous study has examined the effect of stomatal opening on induction in tomatoes (Kaiser *et al*., 2020), which supports our results showing that stomatal induction is important for photosynthetic induction.

A step increase in light intensity induced a tight coupling between *A* and *g*_s_ (Figs 2A, B, 3C), with variations among species. This indicates strong stomatal control of *A*, as demonstrated by substantial stomatal limitations on *A* (Fig. 2F; Supplementary Fig. S3). This phenomenon has been observed in banana (Eyland *et al*., 2021a), cassava (De Souza *et al*., 2020), crop species (McAusland *et al.*, 2016), some tropical tree species (Tinoco-Ojanguren and Pearcy, 1993; Valladares *et al.*, 1997), and various ferns, gymnosperms, and angiosperms (Deans *et al*., 2019), showing that the rate of change in *g*_s_ most limited *A*. On the other hand, Rubisco activation imposed the major limitation on induction in soybean and wheat (Soleh *et al*., 2016b; Taylor and Long, 2017; Salter *et al*., 2019). Thus, the dominant limiting factor, its magnitude, and its duration can vary among species as well as among measurement or cropping environments.

### Smaller stomata with high density result in faster responses of stomatal conductance and photosynthesis

Stomatal density and size differed significantly among species (Fig. 6A, B). Both affect light-induced *g*_s_ response (Hetherington and Woodward, 2003; Drake *et al*., 2012; Raven, 2014; Sakoda *et al*., 2020b). We confirmed that both were correlated with the *g*_s_ kinetics (Fig. 7G, H), indicating that smaller stomatal size and higher stomatal density result in faster responses of *g*_s_ and thus of photosynthetic induction (i.e. *t*50_*A*_; Fig. 7E, F). Our finding is consistent with previous reports that plants with smaller stomata, such as rice, respond faster to environmental stimuli, and could reduce stomatal limitation under dynamic conditions (Ohsumi *et al.*, 2007; Drake *et al.*, 2012; Raven, 2014; McAusland *et al.*, 2016; Kardiman and Ræbild, 2018). The negative relationships between stomatal density and *t*50_*A*_ or *t*50_*g*s_ (Fig. 7F, H) indicate the importance of stomatal density for photosynthetic characteristics during the induction phase.

Stomatal density and size are considered to be adaptive mechanisms in plants in response to environmental stresses, and are known to affect photosynthetic capacity. The quicker response of smaller stomata may result from the higher surface area-to-volume ratio of guard cells, which facilitates faster changes in solute concentrations and guard cell volume (Lawson and Vialet-Chabrand, 2018). In addition, the relationship between *g*_s_ and stomatal aperture is not linear (Vialet-Chabrand *et al.*, 2017) but hyperbolic (Kaiser and Kappen, 1997, 2000), and the influence of stomatal aperture on *g*_s_ decreases rapidly with the magnitude of stomatal opening. Having many small stomata can thus result in a higher *g*_s_ than having fewer large ones (Franks and Beerling, 2009). These two features could explain the faster light-induced *g*_s_ response of smaller stomata during the exponential phase. Collectively, smaller stomata at high density may enable the leaf to achieve rapid *g*_s_ and could be the outcome of selection aimed at improving yields in tomatoes. It is worth noting that our accession of *S. pennellii* had smaller stomatal size and higher stomatal density than either crop variety, providing an intriguing potential source for adaptive variation given its ability to cross with *S. lycopersicum* (Moreels *et al.* 2023).

### Relationship between stomatal characteristics and intrinsic water use efficiency

iWUE is linked to the dynamic range of *A* and *g*_s_ (Fig. 5E, F). Under fluctuating light, *g*_s_ dynamics typically respond more slowly than *A* dynamics (Figs 2A, B, 4B, C). This disparity in dynamics can lead to a lack of synchronization and, consequently, excessive water loss during episodes of fluctuating light. Interestingly, species with high stomatal density, which showed a faster light-induced *g*_s_ response, had lower iWUE_AVE_ values, and species with low stomatal density, which showed a slower light-induced *g*_s_ response, had higher iWUE_AVE_ values (Fig. 7H, L), thus showing a disadvantage of maintaining a high *g*_s_ under fluctuating light conditions. Accelerating stomatal opening caused a pronounced decrease in iWUE_AVE_, as transpiration through the stomata increased faster than CO_2_ assimilation. Thus, it would also be necessary to accelerate the rate of stomatal closure during high light to low light transitions (Lawson *et al*., 2012; Lawson and Blatt, 2014).

The benefit of coordination of *A* and *g*_s_ dynamics for iWUE is evidenced by a recent report that Arabidopsis plants overexpressing the light-gated K^+^ channel (BLINK1) in guard cells had faster stomatal opening and closure under fluctuating light, with resultant greater biomass production at high iWUE (Papanatsiou *et al.*, 2019). Thus, faster stomatal closure during the post-illumination phase, as well as faster opening during the illumination phase, is important to increasing iWUE. This is especially true since a slower reduction in *g*_s_ during the post-illumination phase results in more water loss with no contribution to carbon gain (Lawson and Blatt, 2014). In rice, natural genetic variation in the rate of post-illumination *g*_s_ reduction was associated with variation in drought tolerance (Qu *et al.*, 2016, 2020). Photosynthetic responses following illumination should be investigated to elucidate the role of stomatal response in determining iWUE in tomato.

### Domestication may have limited physiological performance but introgression from wild relatives can enhance it

Natural genetic variation is a fundamental driver of adaptive and neutral evolution. Careful sourcing of adaptive variation from wild species can play a crucial role in crop improvement. Wild tomato species have significant genetic variability, attributed to their diverse habitats across varying climatic gradients (Pease *et al*., 2016). In contrast, tomato cultivars have encountered multiple bottlenecks during their domestication history, resulting in a substantial reduction in genetic diversity (Razifard *et al.*, 2020; Tamburino *et al*., 2020). However, efforts over the past half a century have reintroduced beneficial diversity back into cultivated varieties (e.g. Schouten *et al.*, 2019). Nevertheless, there remains untapped genetic diversity in wild tomato relatives, which can serve as the primary source of high photosynthesis traits for introgression into cultivars. The identification of suitable sources with high photosynthetic capacity is among the most direct strategies for developing high-yield cultivars with enhanced photosynthesis. Therefore, the discovery and characterization of genes associated with high photosynthesis in wild relatives hold promise for both traditional and innovative breeding approaches.

It is generally considered that *S. pimpinellifolium* is the closest wild species to the cultivated tomato, *S. lycopersicum* var. *lycopersicum*, which was derived from *S. lycopersicum* var. *cerasiforme,* which both exists in wild form and was domesticated as cherry tomato (Ranc *et al.*, 2008; Tamburino *et al.*, 2020). Our results clearly show that the photosynthetic performance of our *S. pimpinellifolium* was lower under both steady-state and non-steady-state conditions than that of wild *S. lycopersicum* var. *cerasiforme*, but was similar to those of the two cultivars (Fig. 2A, B). This suggests that during the transition from wild to improved cultivated tomatoes, leaf photosynthetic performance declined. Photosynthesis has been shown to change with domestication and improvement, although not in a uniform way across species (Matesanz and Milla, 2018). Some of that variation has been proposed to be due to progenitors of crops already having higher carbon exchange rates than the average wild species (Milla, 2023). In this system, we are finding that the crop may have inherited its photosynthetic response from the lower performing *S. pimpinellifolium*, as has also been observed in wheat (McAusland *et al*., 2016). Both the closely related wild progenitor species (*S. lycopersicum* var. *cerasiforme*) and more distantly related wild species (e.g. *S. pennellii*) may provide genetic variation to enhance crop performance. Since photosynthesis in landraces of crops has also been shown to be more stress resilient than their improved counterparts (e.g. chili pepper: McCoy *et al.*, 2022), tomato landraces could also be evaluated for adaptive genetic variation. It is apparent that enhancing leaf photosynthesis under both steady-state and non-steady-state conditions represents a valuable objective for improving tomato productivity through both conventional breeding and biotechnology. However, further studies about the degree to which photosynthetic variation is environmentally dependent (i.e. prone to genotype by environment interactions: McCoy *et al.*, 2022) and the relationship between changes in dynamic photosynthesis and yield in cultivated tomato will be needed to evaluate the true benefits of these traits.

Genetic variation within and among plant species for physiological traits has been shown to depend, in part, on the environment of origin (Tiffin and Ross-Ibarra, 2014). Sometimes these patterns can be indicative of local adaptation. For instance, when grown under cool, high elevation conditions, Mexican maize landraces sourced locally tended to have higher photosynthesis, stomatal conductance, and transpiration rates than those from mid and low elevations; those same high elevation landraces had lower physiological performance than the mid and low elevation landraces when grown at hot, low elevations (Pace *et al.*, 2024). Similarly, variation in stomatal conductance and photosynthesis in chili pepper accessions sourced across environmental gradients in Mexico showed a positive linear relationship with precipitation variables (e.g. the seasonality of precipitation, precipitation of the wettest quarter, and total available soil water content; McCoy, 2023). Dittberner *et al.* (2018) also found stomatal size and density to correlate with climatic variation in Arabidopsis from Europe. Accessions from regions with higher temperatures and humidity and lower solar radiation had larger stomata (Dittberner *et al.*, 2018).

Given the different geographical and climatic ranges of crop wild relatives, species may be differentiated for physiological characteristics, in part due to adaptations to the climate in which they are found. Using simple correlations, we found that aspects of the environment of origin of the accessions of wild tomato tested here correlated with their physiological metrics. In particular, we found abaxial stomatal traits, *t*50_*A*_, and *t*50_*g*s_ all correlated with environment of origin (*P<*0.05). Abaxial stomatal size were positively correlated with total number of sunny days per year (Supplementary Figs S7, S8). By contrast, abaxial stomatal density was negatively correlated with sunny day parameters and positively correlated with annual precipitation (Supplementary Figs S7, S9). Moreover, *t*50_*A*_ was positively correlated with elevation and negatively correlated with temperature (Supplementary Figs S7, S10). Finally, *t*50_*g*s_ were positively correlated with total number of sunny days per year (Supplementary Figs S7, S11). It may be worth noting that *A*_max_ had marginally significant (*P*<0.10) correlations with elevation (negative) and temperature (positive) (Supplementary Fig. S7).

These results, which indicate the possibility of natural selection acting on variation in physiological traits, warrant further study with more accessions from promising wild species to clarify if choosing accessions from particular environments of origin might facilitate breeding for enhanced crop photosynthesis. The wild tomato species tested here can be found in multiple climates within their native range (Ramírez-Ojeda *et al.*, 2021). For instance, the crop progenitor *S. pimpinellifolium* can be found in tropical rainforests and savannahs, as well as in arid, hot deserts and steppes, though it is most commonly found in arid, hot deserts. By contrast, *S. pennellii* is more restricted, found only in hot and cold arid deserts (Ramírez-Ojeda *et al.* 2021). Interestingly, *S. lycopersicum* var. *crasiforme* is distributed widely both in South America (where it originated) and in Mexico (where it was domesticated) (Délices *et al.* 2019), so further exploration of intraspecific variation among accessions for photosynthetic traits may be especially fruitful.

### Future challenges for improved dynamic photosynthesis in tomato cultivars

We found significant natural variation among species in the non-steady-state traits *t*50_*A*_, *t*50_*g*s_, *A*_SUM_, and iWUE_AVE_ (Figs 2, 4), indicating variation in tomato that could be exploited to improve photosynthesis in dynamic conditions. Improving the rate of photosynthetic induction would be particularly desirable as it would allow the plant to respond more rapidly to fluctuations in its light environment. Moreover, we found a negative relationship between photosynthetic capacity under steady-state conditions (*A*_max_) and the rate of the induction response (*t*50_*A*_; Fig. 3A), suggesting that both parameters could be improved simultaneously, as has been also reported in wheat (Salter *et al*., 2019). Consequently, improvements in photosynthetic induction have the potential to lead to increased plant productivity, particularly when combined with enhancements in overall photosynthetic capacity (Taylor and Long, 2017). It is worth noting that there are some inconsistencies in the relationship of natural variations between steady-state and non-steady-state photosynthesis under fluctuating light, as there were no significant correlations in soybean, cowpea, and rice (Soleh et al., 2016a; Acevedo-Siaca *et al.*, 2020, 2021; De Souza *et al.*, 2020). Nevertheless, a comprehensive understanding of the genetic mechanisms governing steady-state and non-steady-state photosynthesis under fluctuating light conditions can contribute to optimizing carbon assimilation in crops within field environments across various species.

Recent research has highlighted the potential for significant yield improvements by enhancing photoprotection capacity and non-photochemical quenching (NPQ) dynamics, which involves dissipating excess light energy from the leaf as heat (Kromdijk *et al*., 2016; Hubbart *et al*., 2018). Notably, rapid relaxation of NPQ during transitions from high to low light levels can alleviate limitations on the quantum yield of CO_2_ assimilation, facilitating a swift recovery of photosynthetic efficiency following a reduction in photosynthesis (Kromdijk *et al*., 2016; Murchie and Ruban, 2020). Furthermore, faster stomatal closure under fluctuating light has been shown to lead to increased biomass production with enhanced iWUE (Papanatsiou *et al*., 2019). In summary, improvements in both photosynthetic induction and relaxation, combined with quicker stomatal opening and closure, would result in heightened productivity and improved iWUE. This is particularly significant as photosynthetic induction and relaxation processes occur repeatedly throughout the day owing to fluctuations in light within the crop canopy.

## Conclusion

Our results reveal large genetic variations in the photosynthetic induction response in tomatoes (Figs 2, 4; Supplementry Fig. S1). They show diversity in light-induced *g*_s_ response within tomato species, and that both lower *g*_s_ and slower light-induced *g*_s_ response greatly limit *A* during the induction phase and under simulated natural fluctuating light. The observed diversity in light-induced *g*_s_ response was related to stomatal size and density. Faster dynamics, mediated by smaller stomata at higher density, are likely to be essential criteria for selecting efficient stomatal conductance that accompanies efficient photosynthesis under fluctuating light. Enhancing stomatal responses in dynamic light environments may contribute to the optimization of resource utilization and yields in major crops, thereby informing the development of new crop ideotypes with greater yield potential and increased resilience in the face of future environmental changes.

## Supplementary data

The following supplementary data are available at *JXB* online.

Fig. S1. Photosynthetic parameters under steady-state and non-steady-state conditions in eight wild tomato species and two cherry tomato cultivars.

Fig. S2. Correlations between parameters of photosynthetic induction and stomatal induction.

Fig. S3. Photosynthetic limitation analysis and *C*_i_ throughout photosynthetic induction.

Fig. S4. Photosynthetic parameters under simulated natural fluctuating light for 10 h.

Fig. S5. Correlations between photosynthetic induction parameters and stomatal anatomical characteristics on both abaxial and adaxial sides.

Fig. S6. Heatmap of correlations between physiological parameters, with blue representing positive correlations and red representing negative ones.

Fig. S7. Correlations between physiological measures and environment of accession origin variables.

Fig. S8. Scatterplot of abaxial stomatal size and total yearly days of sun at accession origin by wild tomato species.

Fig. S9. Scatterplot of abaxial stomatal density and either annual precipitation or annual days of sun at accession origin by wild tomato species.

Fig. S10. Scatterplot of *t*50_*A*_ and either elevation or average temperature in °C at accession origin by wild tomato species.

Fig. S11. Scatterplot of *t*50_*g*s_ and annual days of sun at accession origin by wild tomato species.

erae082_suppl_Supplementary_Figures_S1-11

## Data Availability

Data supporting the results can be requested by contacting the corresponding author.

## Abbreviations:

*A*: CO_2_ assimilation rate
*A*_max_: maximum photosynthetic rate
*C*_i_: intercellular CO_2_ concentration
*g*_s_: stomatal conductance
*g*_smax_: maximum stomatal conductance
*g*_sinitial_: stomatal conductance at the beginning of induction
iWUE: intrinsic water use efficiency
*t*50_*A*_: time to reach 50% of maximum photosynthetic rate
*t*80_*A*_: time to reach 80% of maximum photosynthetic rate
*t*90_*A*_: time to reach 90% of maximum photosynthetic rate
*t*50_*g*s_: time to reach 50% of maximum stomatal conductance
*t*80_*g*s_: time to reach 80% of maximum stomatal conductance
*t*90_*g*s_: time to reach 90% of maximum stomatal conductance
