# Supplementary material for: Natural genetic variation in dynamic photosynthesis is correlated with stomatal anatomical traits in diverse tomato species across geographical habitats
Source: J Exp Bot. 2024 Apr 12;75(21):6762–77. doi: 10.1093/jxb/erae082 (PMC11639205; doi:10.1093/jxb/erae082)
Supplement: erae082_suppl_Supplementary_Figures_S1-11 [file erae082_suppl_supplementary_figures_s1-11.pdf]

Title

Natural genetic variation in dynamic photosynthesis is correlated with stomatal anatomical traits in diverse tomato species across geographical habitats

Running title

Natural genetic variation of wild tomatoes in dynamic photosynthesis

Names of all authors

Yugo Yoshiyama[1], Yu Wakabayashi[1], Kristin L. Mercer[1,2], Saneyuki Kawabata[1], Takayuki Kobayashi[3], Toshihito Tabuchi[3], Wataru Yamori[1]*

Names and addresses of the institutions where the work was carried out

[1] Graduate School of Agricultural and Life Sciences, The University of Tokyo, Nishitokyo, Tokyo, Japan

[2] Ohio State University, Department of Horticulture and Crop Science, Columbus, Ohio, USA

[3] Department of Advanced Food Sciences, College of Agriculture, Tamagawa University, Machida, Tokyo, Japan

*Corresponding author: Wataru Yamori, Institute for Sustainable Agro-ecosystem Services, Graduate School of Agricultural and Life Sciences, The University of Tokyo, Tokyo 188-0002, Japan. E-mail: yamori@g.ecc.u-tokyo.ac.jp, ORCID: 0000-0001-7215-4736

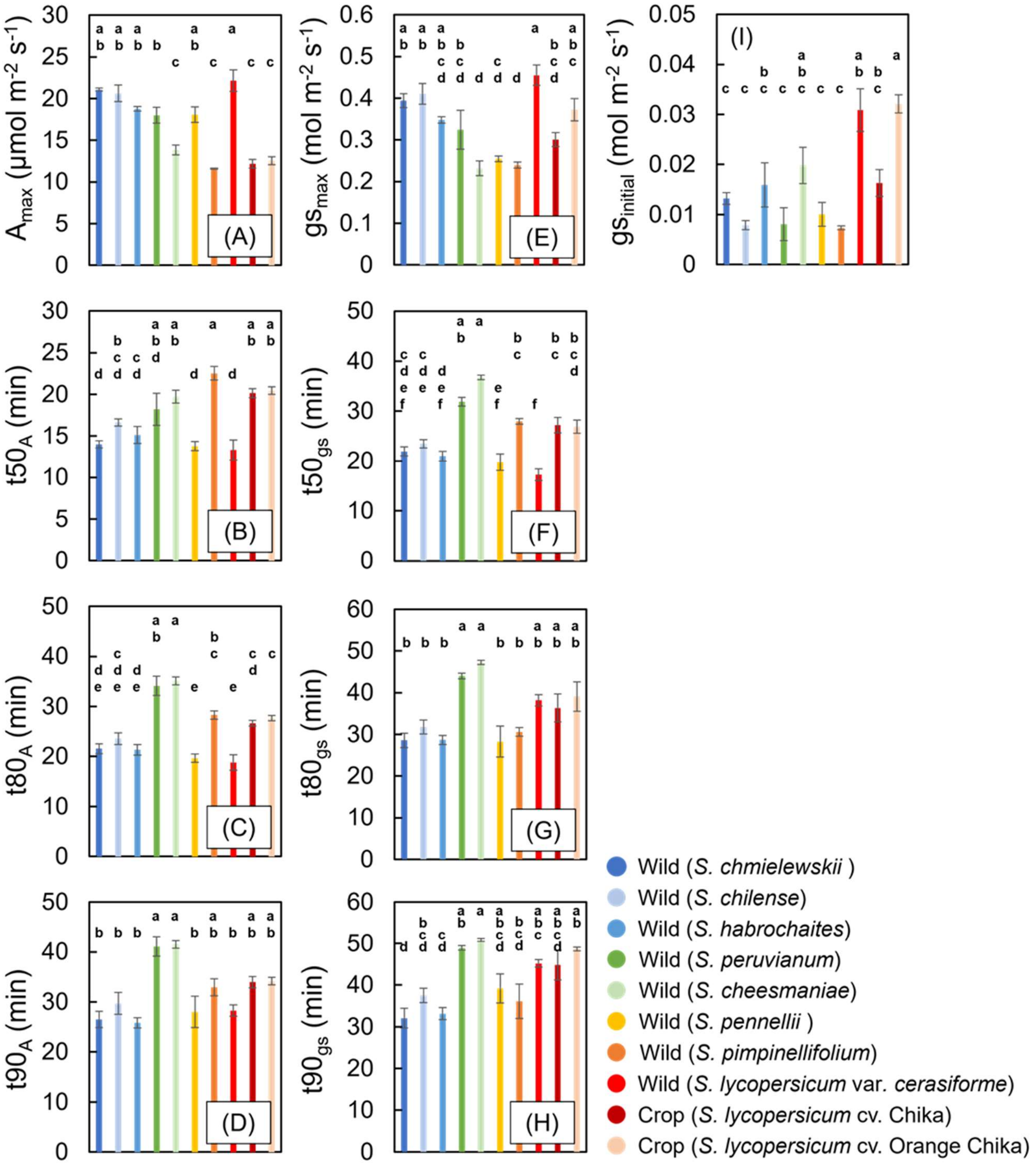

Supplemental Figure 1. Photosynthetic parameters under steady-state and non-steady state conditions in eight wild tomato species and two cherry tomato cultivars. Parameters were acquired from photosynthetic induction (Fig. 2). Data are mean ± SE, n = 4–6. Bars with the same letter are not significantly different at $P < 0.05$ by Tukey's test.

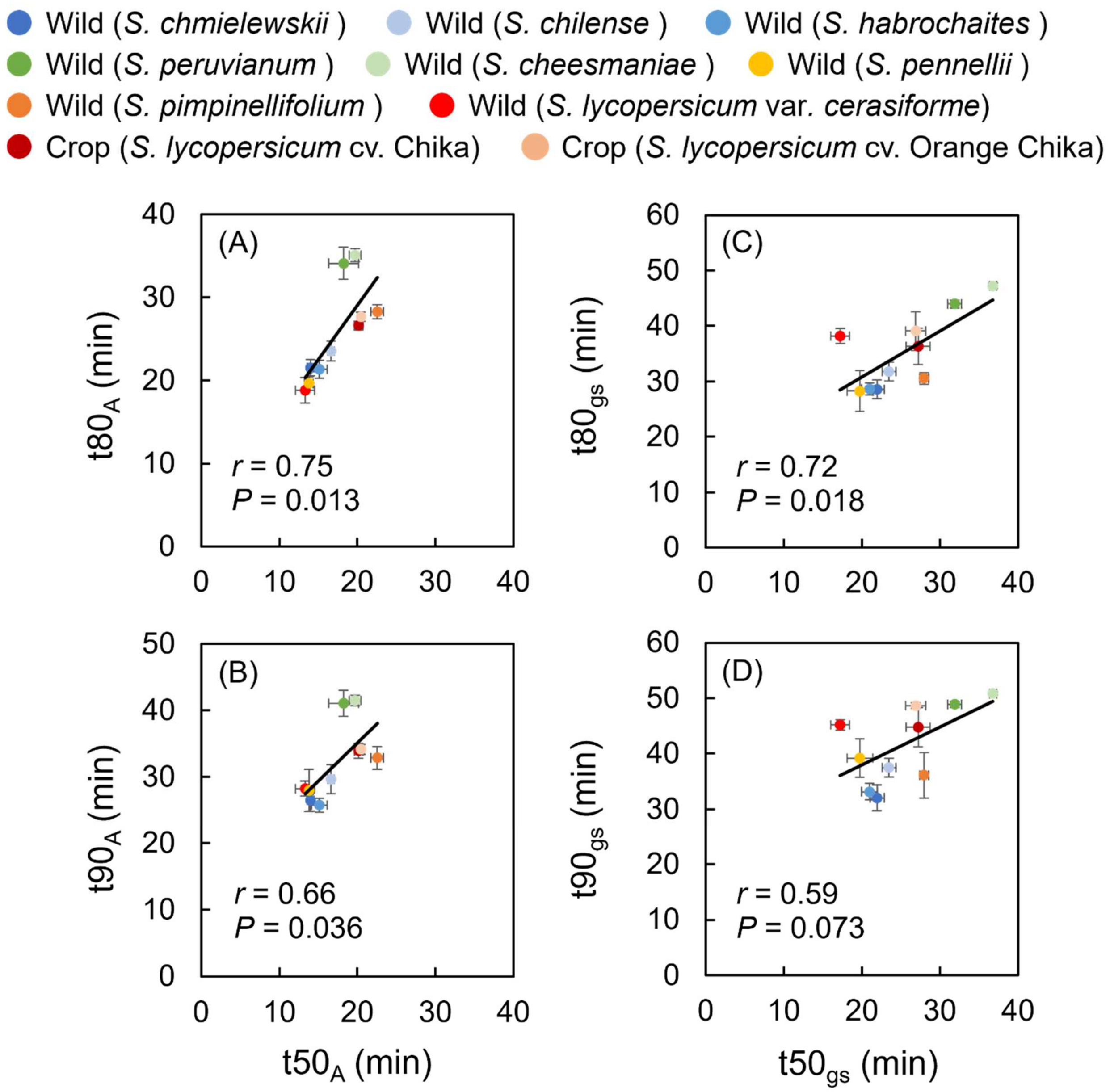

Supplemental Figure 2. Correlations of $t50_A$ with (A) $t80_A$ and (B) $t90_A$; and of $t50_{gs}$ with (C) $t80_{gs}$ and (D) $t90_{gs}$. Data are mean ± SE, n = 4–6. Solid lines indicate significant correlations. Solid lines indicate significant correlations. See text for abbreviations.

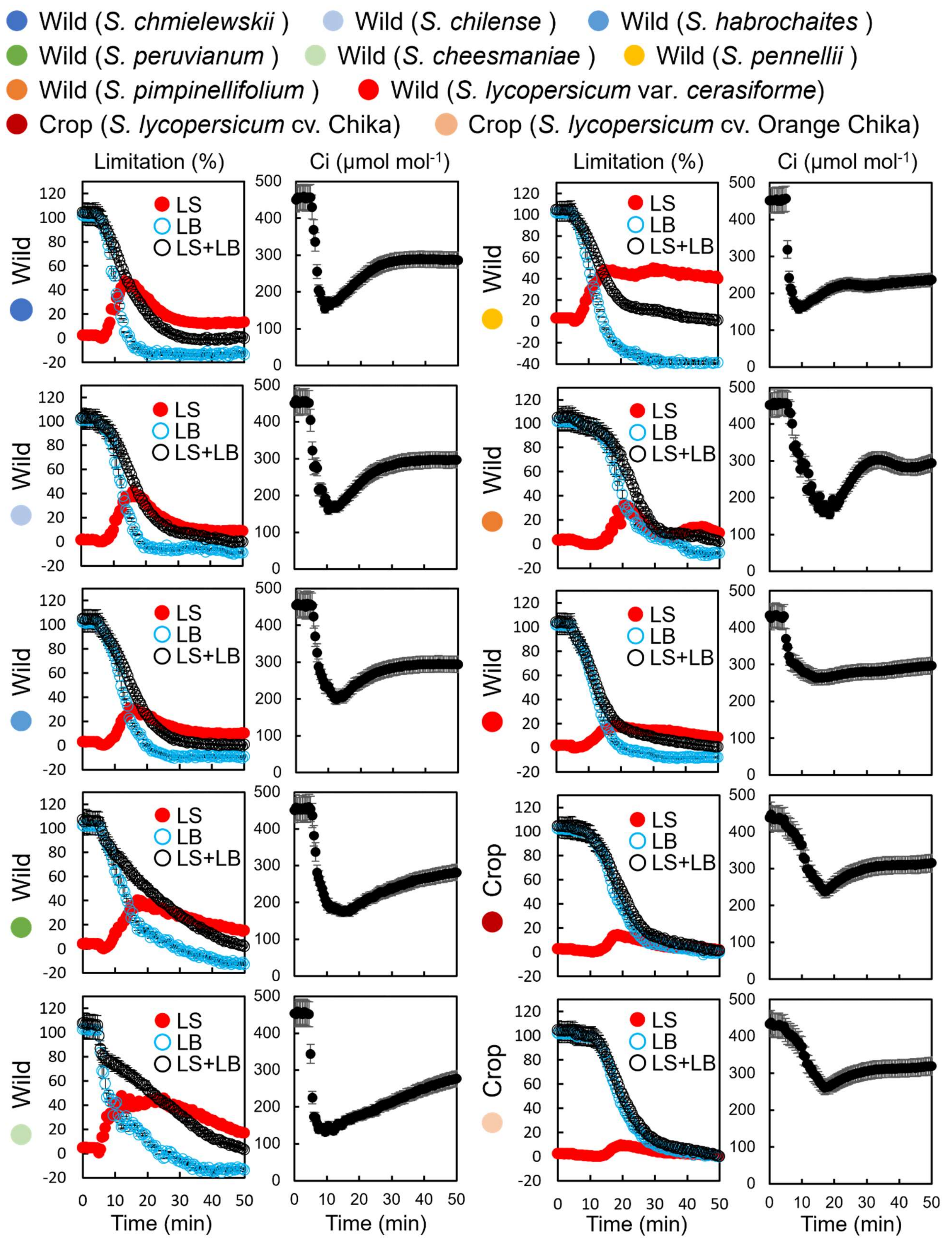

Supplemental Figure 3. Photosynthetic limitation analysis and C_i throughout photosynthetic induction. L_S, stomatal limitation; L_B, Biochemical limitation. $L_S = \{(A_{Ca}^* - A) / (A_f - A_i)\} \times 100$; $L_B = \{(A_f - A_{Ci}^*) / (A_f - A_i)\} \times 100$, where A_{Ca}^* and A_{Ci}^* are A corrected for changes in transient C_i. Data are mean ± SE, n = 4–6.

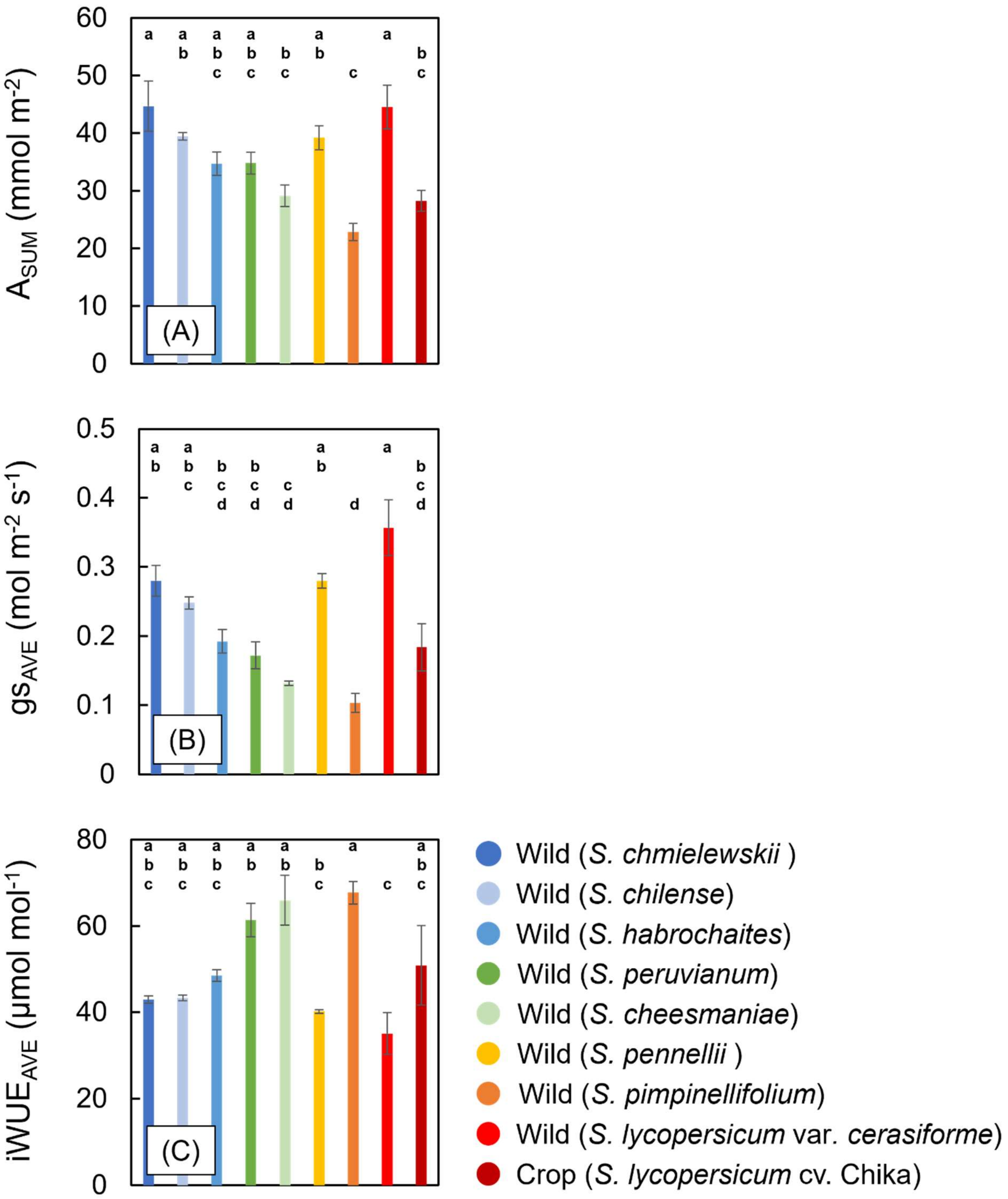

Supplemental Figure 4. Photosynthetic parameters under simulated natural fluctuating light for 10 h. Parameters were acquired from diurnal irradiation (Fig. 4). Data are mean ± SE, n = 4–6. Bars with the same letter are not significantly different at $P < 0.05$ by Tukey's test.

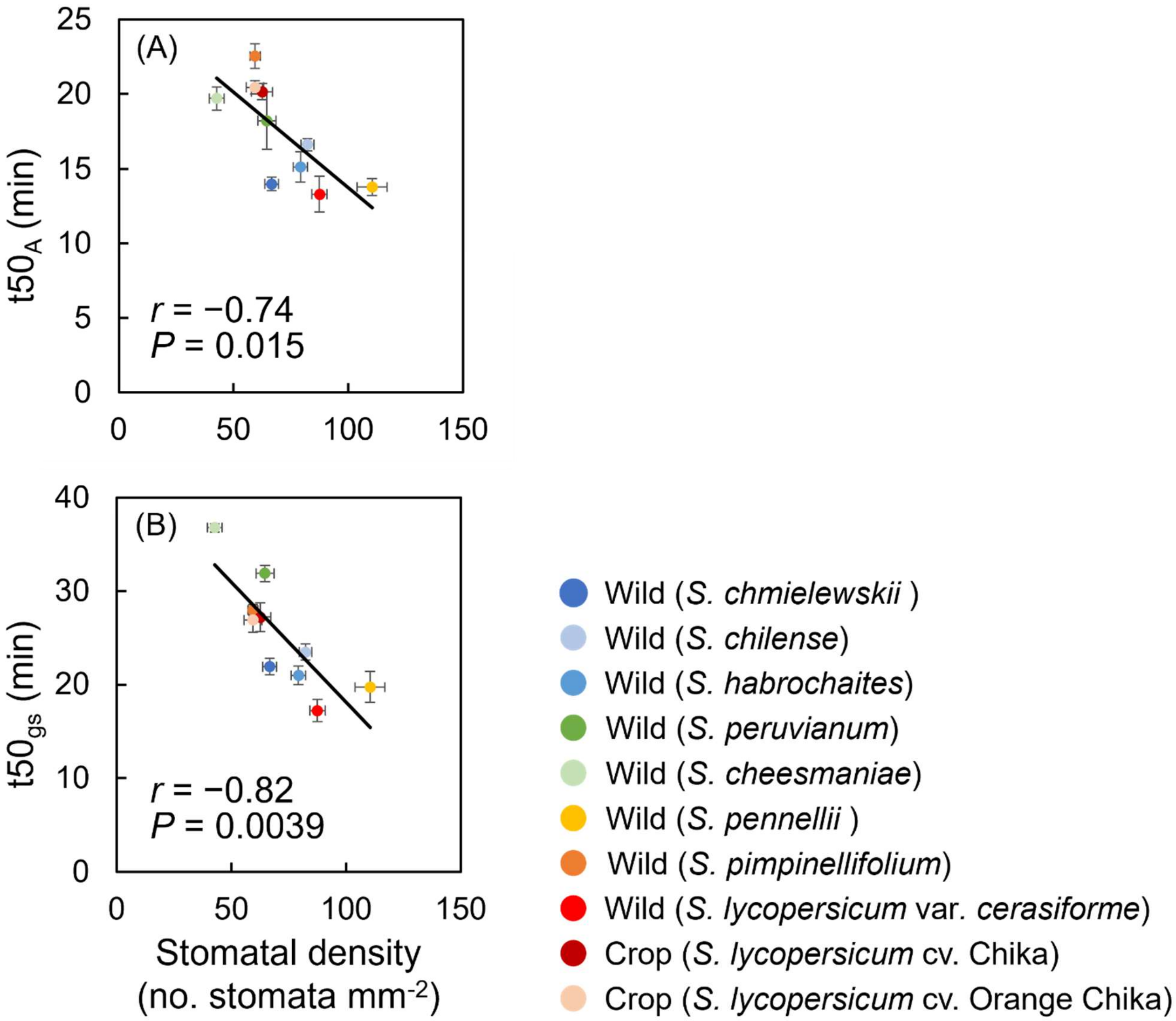

Supplemental Figure 5. Correlations between photosynthetic induction parameters and stomatal anatomical characteristics on both abaxial and adaxial sides. Data are mean ± SE, n = 4–6. Solid lines indicate significant correlations.

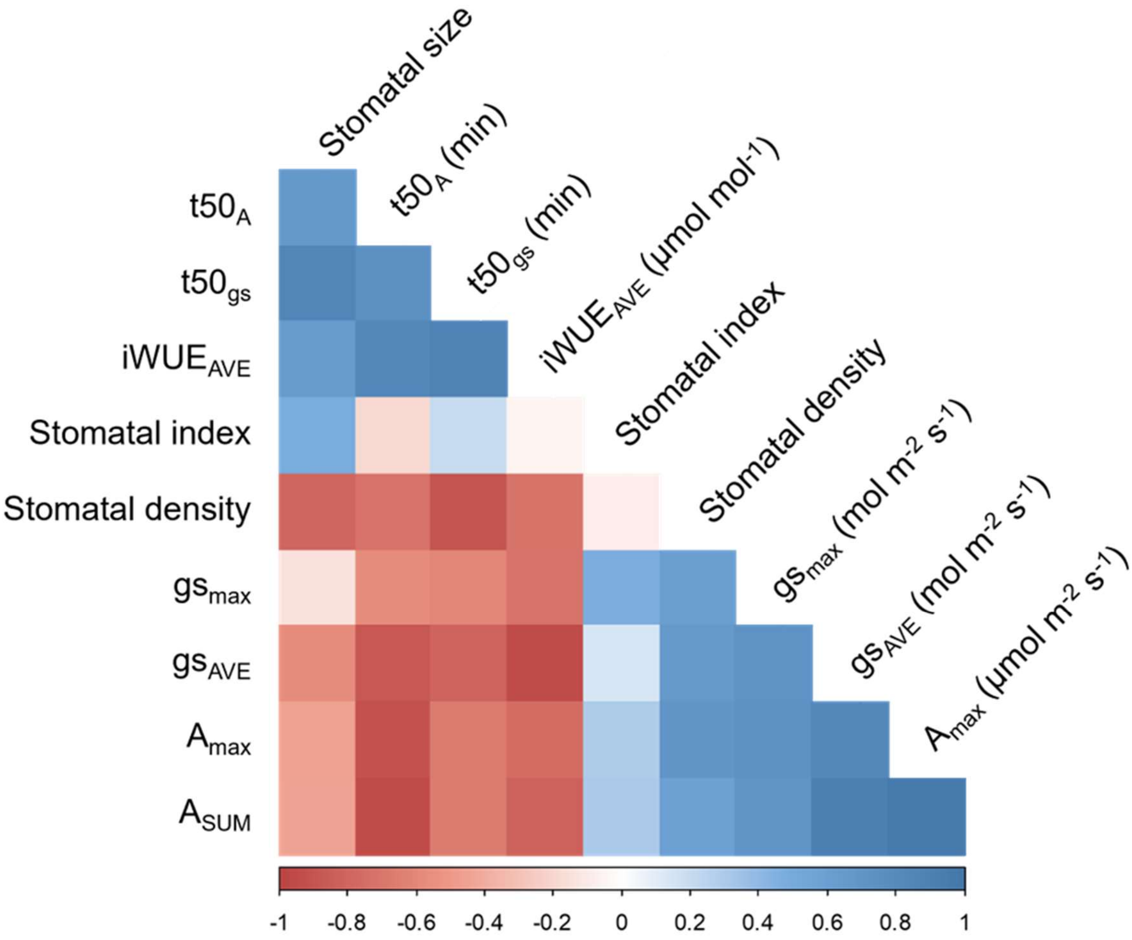

Supplemental Figure 6. Heatmap of correlations between physiological parameters with blue representing positive correlations and red representing negative ones. See text for abbreviations.

Environmental variable		Physiological measure: Amax	gsmax	ASUM	abaxial stoma size	abaxial stoma density	$t50_A$	$t50_{gs}$
Elevation (m)	r	-0.337	-0.166	-0.299	-0.195	0.071	**0.369**	-0.089
	P	0.059	0.364	0.122	0.2846	0.7013	0.038	0.628
	n	32	32	28	32	32	32	32
Average temperature (°C)	r	0.313	0.156	0.279	-0.085	0.223	**-0.379**	-0.119
	P	0.081	0.395	0.151	0.6439	0.2202	0.033	0.518
	n	32	32	28	32	32	32	32
Annual precipitation (mm)	r	0.161	0.171	0.052	-0.256	**0.459**	-0.243	-0.248
	P	0.378	0.348	0.793	0.1571	0.0082	0.18	0.17
	n	32	32	28	32	32	32	32
Annual days with sun (num.)	r	-0.171	-0.151	-0.196	**0.538**	**-0.514**	0.251	**0.505**
	P	0.35	0.409	0.317	0.0015	0.0026	0.166	0.003
	n	32	32	28	32	32	32	32

	p<0.05	negative r
	0.10>p.0.05	negative r
	p<0.05	positive r
	0.10>p.0.05	positive r

Supplementary Figure 7. Correlations between physiological measures and environment of accession origin variables. Pearson's correlation coefficients (r), p-values (P), and number of observations used to calculate correlations included. See text for abbreviations of physiological measures.

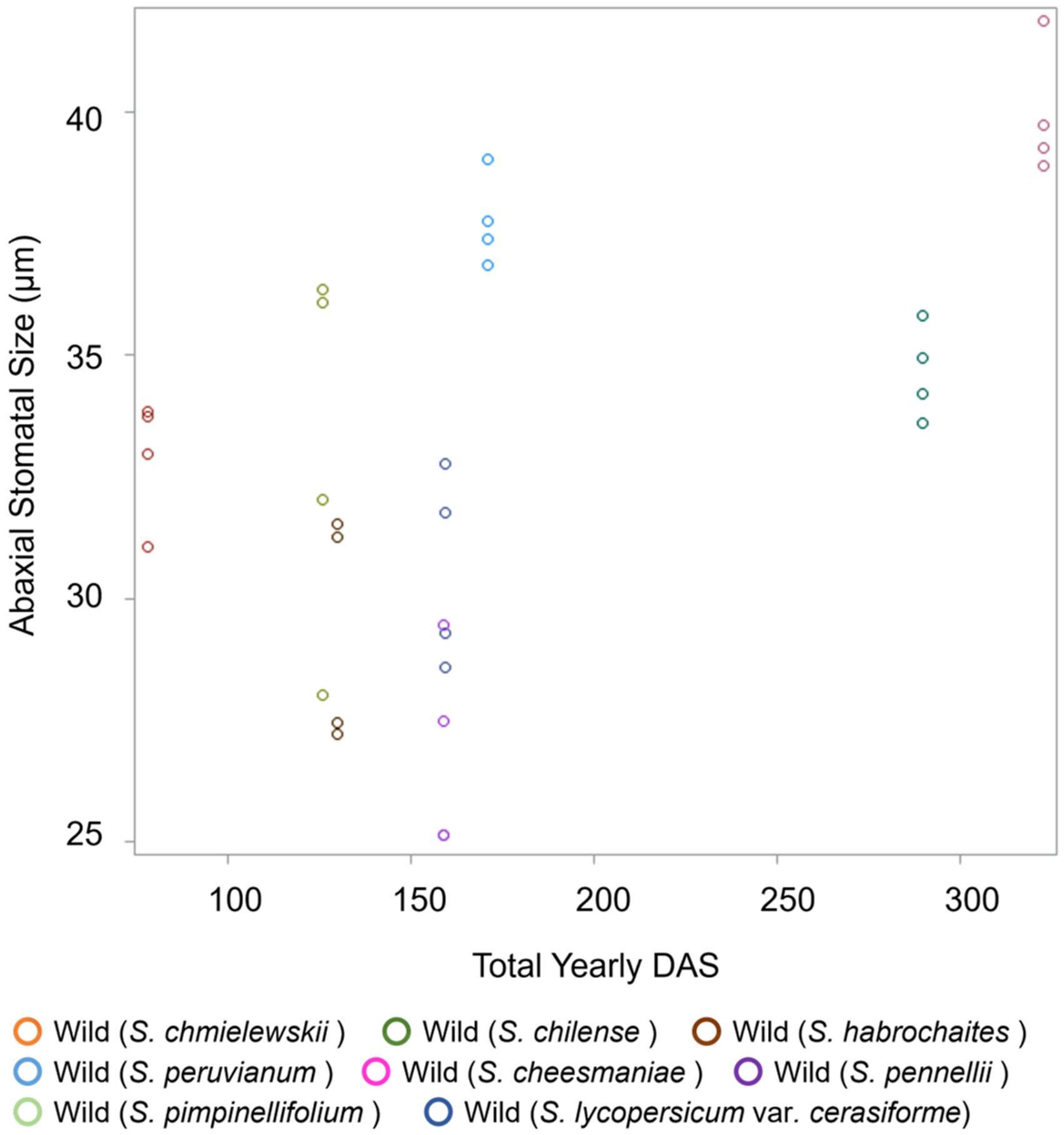

Supplementary Figure 8. Scatterplot of abaxial stomatal size and total yearly days of sun at accession origin by wild tomato species. All species are in the Solanum genus.

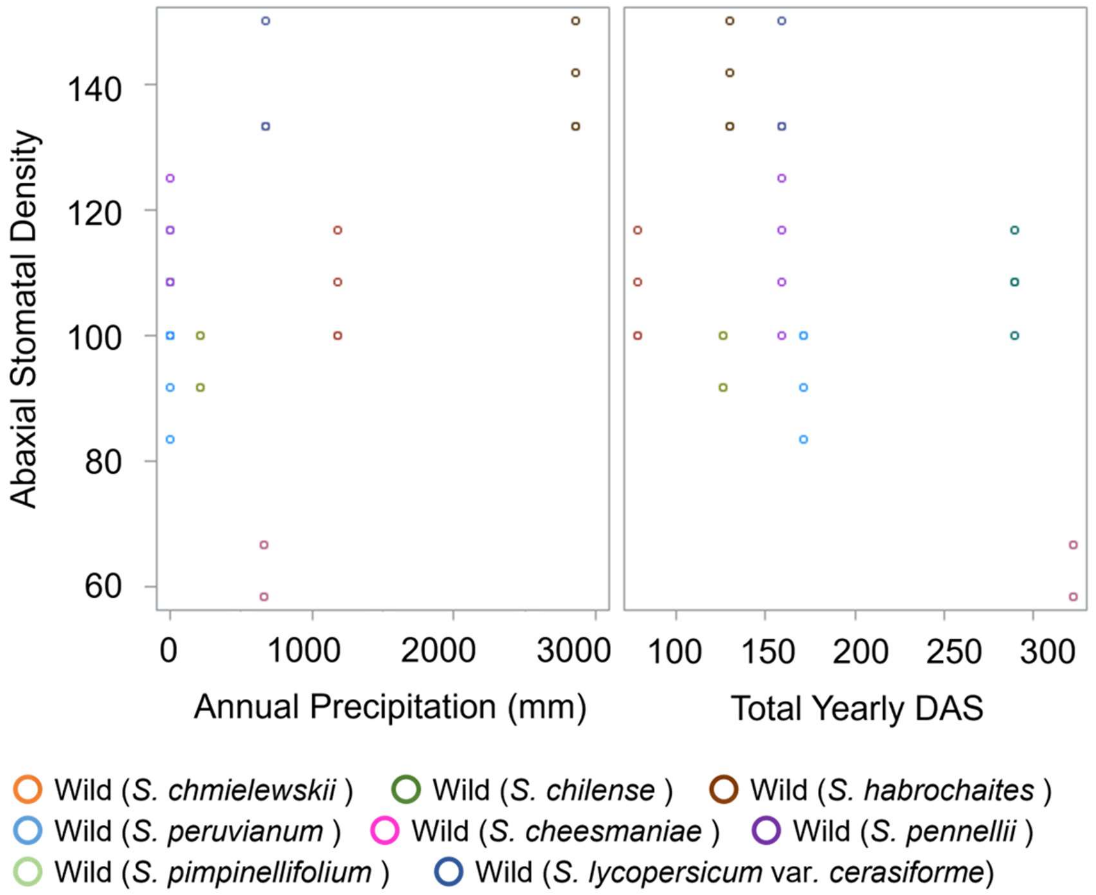

Supplementary Figure 9. Scatterplot of abaxial stomatal density (ab_stom_dens) and either annual precipitation (annual_precip_mm) or annual days of sun (total_yearly_DAS) at accession origin by wild tomato species. All species are in the Solanum genus.

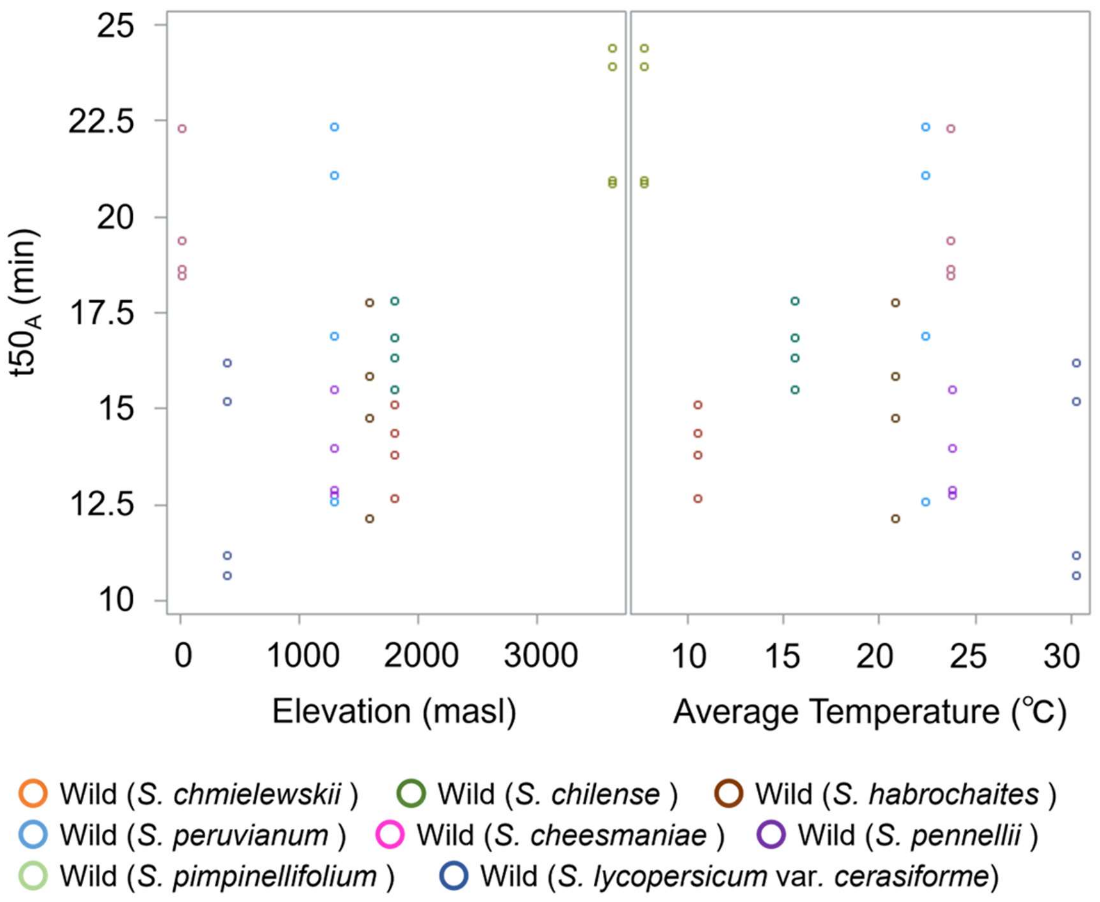

Supplementary Figure 10. Scatterplot of t50A (At50) and either elevation (elev_masl) or average temperature in ° C (avg_temp_C) at accession origin by wild tomato species. All species are in the Solanum genus.

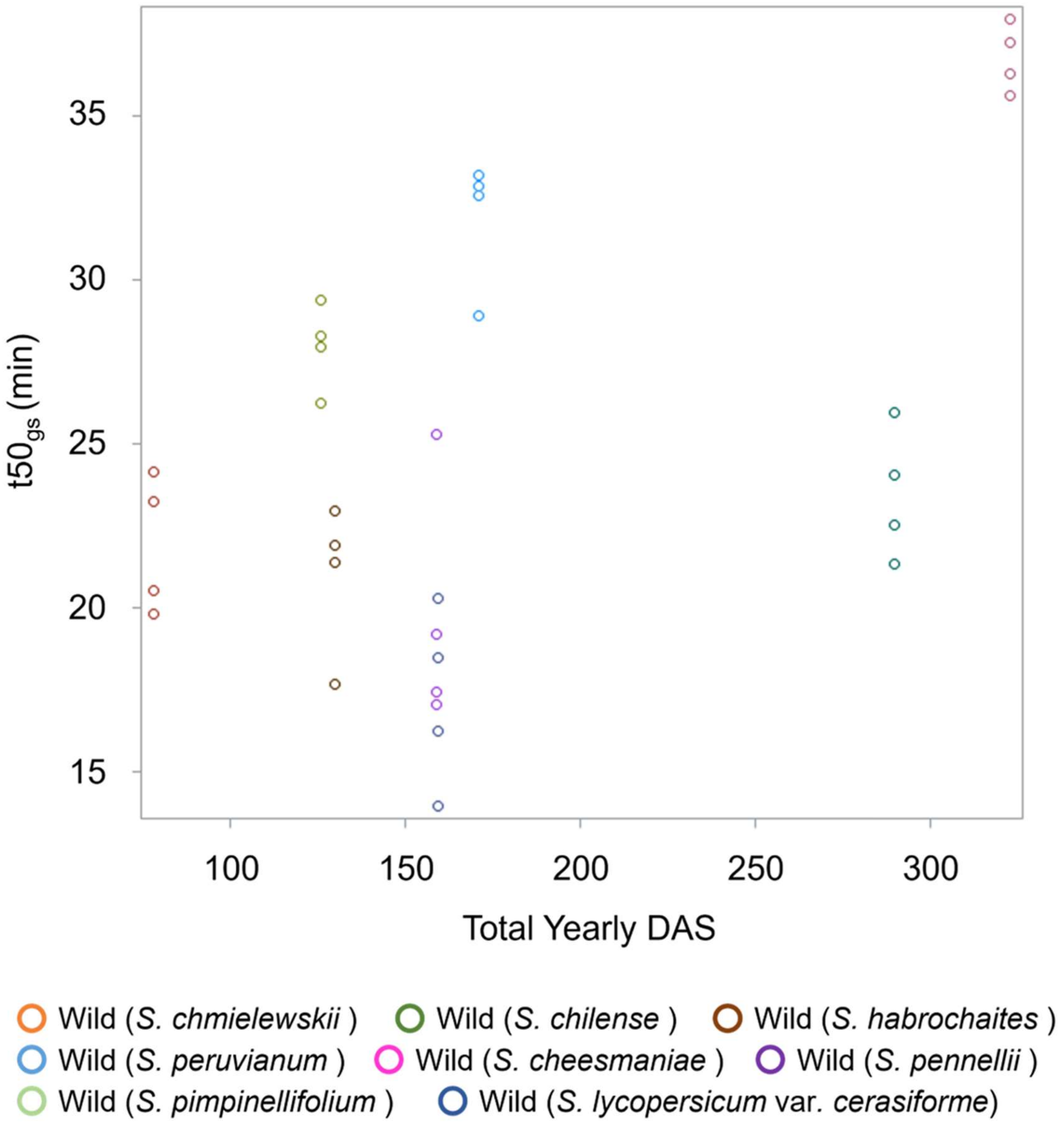

Supplementary Figure 11. Scatterplot of t50gs (gst50) and annual days of sun (total_yearly_DAS) at accession origin by wild tomato species. All species are in the Solanum genus.
